# The ideas of people referred to neurologists about managing their headaches: A qualitative study

**DOI:** 10.1186/1129-2377-1-S14-P203

**Published:** 2013-02-21

**Authors:** F Nadeem, A Noble, L Ridsdale, M Morgan

**Affiliations:** 1 Kings College London, UK

## Introduction

Headache is the commonest reason for General Practitioner (GP) referrals to neurologists, accounting for 25% of all referrals. Those that are referred, however, constitute only 2% of patients who consult GPs with headache. Previous research has suggested that referred patients are more fearful and anxious about their symptoms than those managed without referral. GPs described pressure to refer, often for a brain scan. We now report patients' perspectives.

## Aims/objectives

The aim of this study was to explore the view of people consulting GPs with headache who were referred to neurologists.

## Methods

A qualitative study using semi-structured interviews with nineteen adults aged 23-63, referred by their GPs to neurologists for primary headaches. Audio-recorded interviews were transcribed and analysed thematically.

## Results

Participants described recurring concerns about secondary organic causes for headache, like a brain tumour. They described their headaches as stressful and a vicious cycle, with further headaches occurring. Some reported catastrophic fears, leading them to attend A&E. Many believed they needed a brain scan, and over half had had a scan, all of which were normal. Many reported dissatisfaction with care and use of alternative therapies.

## Conclusion

People referred to neurologists for headache described fear and distress, particularly about the possibility of a brain tumour. GPs now have open access to scanning. This may relieve physical concerns. Interventions to address health-related anxiety may help some consulters for headache too.
